# Effects of secreted frizzled-related protein 1 on proliferation, migration, invasion, and apoptosis of colorectal cancer cells

**DOI:** 10.1186/s12935-018-0543-x

**Published:** 2018-03-27

**Authors:** Zhongchuan Wang, Rujia Li, Yongshan He, Shiyong Huang

**Affiliations:** Department of Colorectal Surgery, Xinhua Hospital Affiliated to Shanghai Jiaotong University, School of Medicine, No. 1665 Kongjiang Road, Shanghai, 200092 China

**Keywords:** Apoptosis, Colorectal cancer, Invasion, Migration, Proliferation, SFRP1

## Abstract

**Background:**

Secreted frizzled-related protein 1 (SFRP1) is a member of the SFRPs family that modulates the Wnt signal transduction pathway. Recent studies have showed down-regulation of SFRP1 expression in colorectal cancer (CRC). We aimed to evaluate the effect of SFRP1 on the proliferation, migration, invasion and apoptosis of CRC cells in vitro.

**Materials and methods:**

We used real-time fluorescence quantification (RT-PCR) and Western blotting to detect SFRP1 expression in CRC, pericarcinomatous tissues and CRC cell lines. We assessed the influence of overexpression and knockdown of SFRP1 on CRC cell proliferation, migration, invasion, and apoptosis, Western blotting was used to evaluate protein levels of Wnt, β-catenin, and apoptosis-related proteins.

**Results:**

The expression of SFRP1 was significantly decreased in CRC tissues. Among the six CRC cell lines (sw-480, sw1116, caco-2, ht-29, colo-205, and hct-116), RT-PCR revealed that sw1116 cells had the lowest expression of SFRP1, while caco-2 cells had the highest SFRP1 expression. SFRP1 overexpression in sw1116 cells significantly suppressed cell proliferation while SFRP1 knockdown in caco-2 cells significantly increase the cell proliferation. In addition, overexpression of SFRP1 in sw1116 cells remarkedly suppressed cell migration and invasion, whereas knockdown of SFRP1 in caco-2 cells resulted in significant enhancement of migration and invasion. Furthermore, SFRP1 overexpression in sw1116 cells promoted cell apoptosis. Western blotting showed that SFRP1 overexpression significantly decreased the protein levels of Wnt, β-catenin and apoptosis-related proteins, including MMP2, MMP9, Twist, CDK1, TGF, and Bcl2.

**Conclusion:**

Our results demonstrate that SFRP1 suppresses cell proliferation, migration and invasion, and promotes apoptosis in CRC cells.

## Background

Colorectal cancer (CRC) is one of the leading causes of morbidity and mortality worldwide, accounting for about 700,000 deaths per year [1]. Despite extensive research efforts over the past two decades to identify factors contributing to the development and progression of CRC, the pathogenesis of CRC remains largely unknown. CRC is thought to be a complex disease that is caused by interactions between genetic, epigenetic and environmental factors [2]. Given the high prevalence and significant economic burden of CRC, a better understanding of the molecular mechanisms involved in colorectal tumorigenesis may help improve treatment for CRC patients.

The Wnt-secreted proteins are a family of glycosylated lipid-modified proteins that play an important role in development, tissue homeostasis, cell differentiation and cell proliferation [3]. Aberrant activation of the Wnt signaling pathway have been implicated in the pathogenesis of various types of human tumors including CRC [4]. Wnt proteins bind to Frizzled transmembrane receptors and initiates a complex signalling cascade, which leads to stabilization of an intracellular transcriptional coactivator called β-catenin. After translocation into the nucleus, β-catenin generates a transcriptionally active complex with T cell factor/lymphoid enhancing factor (TCF/LEF) transcription factors to induce Wnt target gene transcription. A family of secreted proteins called secreted Frizzled-related proteins (SFRPs) has recently been identified as extracellular regulators of the Wnt signaling pathway [5]. SFRPs possess a cysteine-rich domain (CRD) homologous to the frizzled receptors. They can inhibit Wnt signaling by sequestering Wnts through their CRD or by forming inactive complexes with the frizzled receptors [5].

SFRP1 is an antagonist of Frizzled receptors and Wnt pathway activation; its mRNA expression has been detected in various tissues such as kidney, heart, colon, breast and brain [6]. The *SFRP1* gene is located at chromosome 8p12-p11.1, within a common deleted region associated with the development of many human tumors [6]. Recent studies have demonstrated down-regulation of SFRP1 in CRC [7–9]. Using semiquantitative analysis by real-time polymerase chain reaction (PCR), the study by Caldwell et al. showed that SFRP1 mRNA expression was down-regulated in CRC cases in comparison to matched normal large bowel mucosa [7]. In agreement with their findings, Qi and coworkers found that the levels of SFRP1 mRNA expression were markedly reduced or silenced in colorectal carcinomas and adenomas compared with the normal mucosa, and the reduced SFRP1 expression was significantly associated with aberrant hypermethylation of the *SFRP1* gene [8]. In addition, loss of SFRP1 protein expression in human CRC tissue was found to be associated with deep invasion and high TNM stage [9]. Moreover, In vitro studies showed that overexpression of *SFRP1*, *SFRP2* and *SFRP5* in colorectal cancer cells resulted in decreased levels of overall cytoplasmic and nuclear β-catenin and decreased colony formation, suggesting a tumor-suppressing effect of *SFRP1* [10].

Although frequent hypermethylation of the *SFRP1* promoter and down-regulation of SFRP1 expression have been observed in CRC, the role of SFRP1 in colorectal tumorigenesis remains poorly understood. In the present study, we aimed to investigate the effects of SFRP1 on proliferation, migration, invasion and apoptosis of CRC cells in vitro and the underlying mechanism.

## Materials and methods

### Clinical samples

Paired tumor and adjacent normal tissue samples were collected at the time of dissection from patients with CRC at the Xinhua Hospital Affiliated to Shanghai Jiaotong University. All tumor tissues were histologically confirmed. The tissue biopsies were frozen and stored at − 80 °C until analysis. The study was performed according to the ethical standards of the revised version of Helsinki Declaration. The research ethics committee of the hospital approved the study.

### Cell treatment

The sw-480, sw-1116, caco-2, ht-29, colo-205, and hct-116 cell lines were purchased from ATCC (Virginia, USA), and cultivated in RPMI 1640 with 10% (v/v) fetal bovine serum (FBS) (Invitrogen, Carlsbad, CA). Cells were incubated in a humidified atmosphere (5% CO_2_ and 37 °C). The ORF plasmid of SFRP1 was obtained from GeneCopoeia. pEZ-Lv201 Vector was used to build an over-expression system of SFRP1. Negative control was pEZ-Lv201, and control was the normal sw-1116 cells. All lentiviral particles were generated by following a standardized protocol using highly purified plasmids, Endo Fectin-Lenti™ and Titer Boost™ reagents (FulenGen, Guangzhou, China). The lentiviral transfer vector was co-transfected into cells with Lenti-Pac™ HIV packaging mix (FulenGen, Guangzhou, China). Lentivirus-containing supernatant was harvested, clarified, and stored at − 80 °C 48 h after transfection. Double-stranded RNAs (dsRNA) targeting the *SFRP1* gene and complementary dsRNA were synthesized (ReiBo Biotech, China). siRNA targeting *SFRP1* (5′-GGCCAUCAUUGAACAUCUCtt-3′ and 5′-GAGAUGUUCAAUGAUGGCCtt-3′) and a negative control termed siRNA_NC (5′-UUCUCCGAACGUGUCACGUtt-3′ and 5′-ACGUGACACGUUCGGAGAAtt-3′) were also synthesized in this study. Cells were seeded at a density of 5 × 10^5^ cells per well of six-well plates with DMEM plus 10% FBS (containing no antibiotics) overnight. Transfection was carried out with OPTI-MEM serum-free medium and Lipofectamine 2000 reagent (final siRNA concentration: 50 or 100 nM).

### RT-PCR

Reverse transcription of mRNA from tumor, pericarcinomatous tissues, and the cell lines was carried out in a final volume of 100 µl containing 400 ng total RNA using the high capacity cDNA Archive kit (Applied Biosystems). SFRP1 and GAPDH mRNA levels were determined by RT-PCR; the primers were described in Table 1. Reactions were performed in 50 µl volumes containing SYBR Green PCR master mix (Perkin-Elmer Biosystems). Real-time PCR was performed using a GeneAmp PCR System 9600 (Perkin-Elmer Biosystems) in 96-well optical plates. Thermal cycling conditions were as follows: 50 °C for 2 min, 95 °C for 10 min, followed by 40 cycles of 95 °C for 30 s, 60 °C for 30 s, and 72 °C for 2 min. Data were collected using the ABI analytical thermal cycler. The delta–delta Ct method was used to determine the RNA expression.

**Table 1:** Specific primers designed for the SFRP1 and GAPDH cDNA clone

Gene	Primers	Length (bp)	Tm (°C)
SFRP1	5-CTCAACAAGAACTGCCACGC-3	135	58.4
			58.4
	5-CTCGTTGTCACAGGGAGGA-3		
GAPDH	5-CAAGTTCAACGGCACAGTCA-3	122	60.04
			60.04
	5-CACCCCATTTGATGTTAGCG-3		

### Cell proliferation assays

A cell proliferation assay was conducted with MTT kit (Sigma) according to the manufacturer’s instruction. For the colony formation assay, 500 cells were placed into each well of six-well plate and maintained in media containing 10% FBS for 2 weeks. Colonies were fixed with methanol and stained with 0.1% crystal violet (Sigma) in PBS for 15 min. Colony formation was judged by counting the number of stained colonies in three randomly selected fields using an inverter microscope. Triplicate wells were measured in each treatment group.

### Migration and invasion assay

Cell migration assay was performed using Transwell Permeable Support (Corning Incorporated, Corning, NY, USA). After transfection, caco-2 and sw1116 cells were carefully transferred on the top chamber of each transwell apparatus at a density of 1 × 10^6^ cells/ml (100 µl per chamber). Cells were allowed to migrate for 24, 48 and 72 h at 37 °C. Those penetrating to the bottom side of the membrane were then fixed in methanol, stained with hematoxylin, and counted using a microscope. Cell invasion was analyzed by using Cultrex 24-well BME Cell Invasion Assay (Trevigen Inc., Gaithersburg, MD, USA) according to standard procedures. Briefly, after transfection, 10^3^ cells in 100 µl serum-free media were seeded into the upper wells pre-coated with Matrigel basement extract, and 500 µl of media were added into the bottom wells. After 24, 48 and 72 h of incubation at 37 °C in a CO_2_ incubator, the non-invasive cells on the upper surface were removed, whereas the cells migrating to the lower surface were fixed in 500 µl of Cell Dissociation Solution/Calcein-AM, incubated at 37 °C in a CO_2_ incubator for 1 h and quantified by fluorimetric analysis (485 excitation, 520 nm emission).

### Western blot analysis

Total cellular protein was isolated using 1% PMSF and RIPA lysis buffer (50 mM Tris–HCl pH 7.4, 150 m MNaCl, 1% NP-40, 0.1% SDS). Protein lysates were mixed with SDS-PAGE loading buffer and boiled at 100 °C for 5 min prior to electrophoresis. Samples were resolved on SDS-PAGE gels and transferred onto polyvinylidene fluoride (PVDF) membranes (Millipore, USA). After incubation for 1 h with blocking buffer at room temperature, the membranes were incubated with rabbit anti-mouse polyclonal primary antibodies (GAPDH, SFRP1, Wnt, β-catenin, MMP2, PCNA, Twist, caspase3, MMP9, CDK1, NFAT, TGF, Bax, Bcl2 and Ap1; all from ABGENT, USA) at a dilution of 1:1000 overnight. Incubation with secondary antibodies was performed at room temperature for 1 h prior to detection with ECL reagent (Advansta, USA). The bands were obtained by GeneGnome 5 (Synoptics Ltd., UK).

## Results

### Downregulation of SFRP1 in primary human CRC and cell lines

**Fig. 1:**
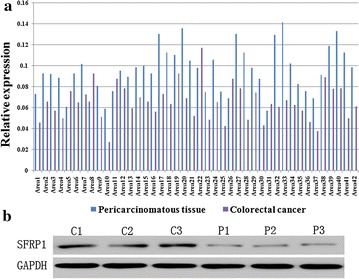
SFRP1 mRNA expression was down-regulated in primary human colorectal cancer. **a** Relative mRNA expression of SFRP1 by RT-PCR. **b** Relative protein expression of SFRP1 by Western blot. RT-PCR was performed on 42 paired colorectal cancer and pericarcinomatous tissues. GAPDH was used as a control for cDNA synthesis. Blue column and purple column indicated samples of pericarcinomatous tissues and colorectal cancer, respectively. SFRP1 mRNA expression was down-regulated in the colorectal cancer compared to pericarcinomatous tissues. The results mentioned above were confirmed by Western blot analysis of three random selected paired samples

**Fig. 2:**
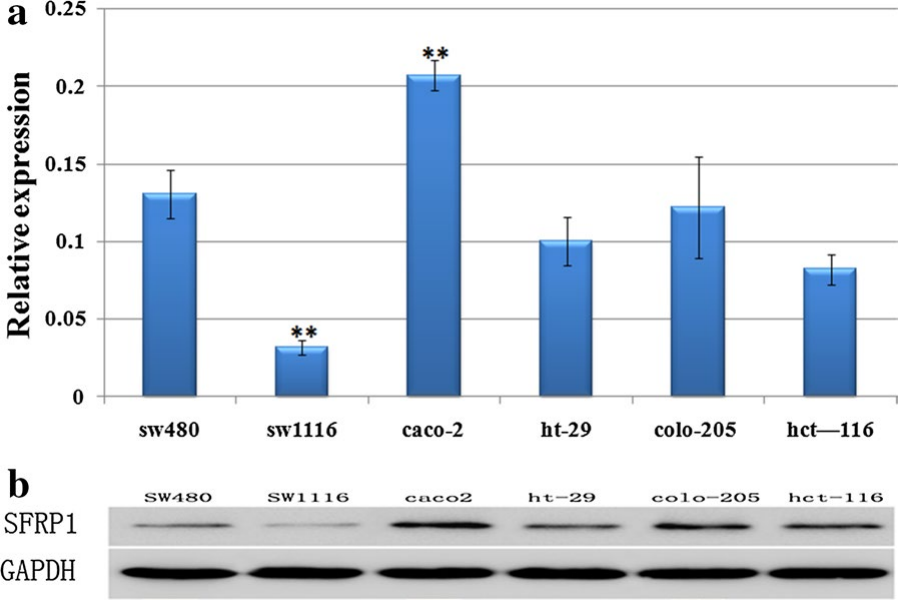
Expression of SFRP1 in six human colorectal cancer cell lines. **P < 0.01, compared with sw480 cell line. **a** Relative mRNA expression of SFRP1 by RT-PCR. **b** Relative protein expression of SFRP1 by Western blot. RT-PCR and western blot were performed on six human colorectal cancer lines. GAPDH was used as a control for cDNA synthesis. SFRP1 mRNA expression was significantly reduced in human sw1116 cells, while it was significantly overexpressed in human caco-2 cells

In order to investigate SFRP1 expression, SFRP1 cDNA was selected for verification of differential expression by RT-PCR. Using specific primers designed for the SFRP1 cDNA clone (Table 1), RT-PCR was performed on 42 paired CRC and pericarcinomatous tissues. The RT-PCR analysis showed that compared with pericarcinomatous tissues, SFRP1 expression was markedly decreased in CRC tissues, which was further confirmed by Western blot analysis (Fig. 1) SFRP1 expression was then assessed by RT-PCR in six CRC cell lines (sw-480, sw1116, caco-2, ht-29, colo-205, and hct-116); GAPDH was used as a control for both cDNA quality and efficiency of the PCR amplification. As shown in Fig. 2, the SFRP1 transcript was detectable in all CRC cell lines. The highest mRNA levels of SFRP1 were found in the caco-2 cell line, whereas the lowest SFRP1 expression was observed in the sw1116 cell line, indicating that SFRP1 mRNA expression in sw1116 cells was more similar to that of CRC. Western blot analysis confirmed decreased SFRP1 protein expression in CRC cell lines (Fig. 2); the lowest SFRP1 protein expression was found in sw1116 cells.

### Effects of overexpression and knockdown of SFRP1 on apoptosis, migration and invasion of CRC cells

**Fig. 3:**
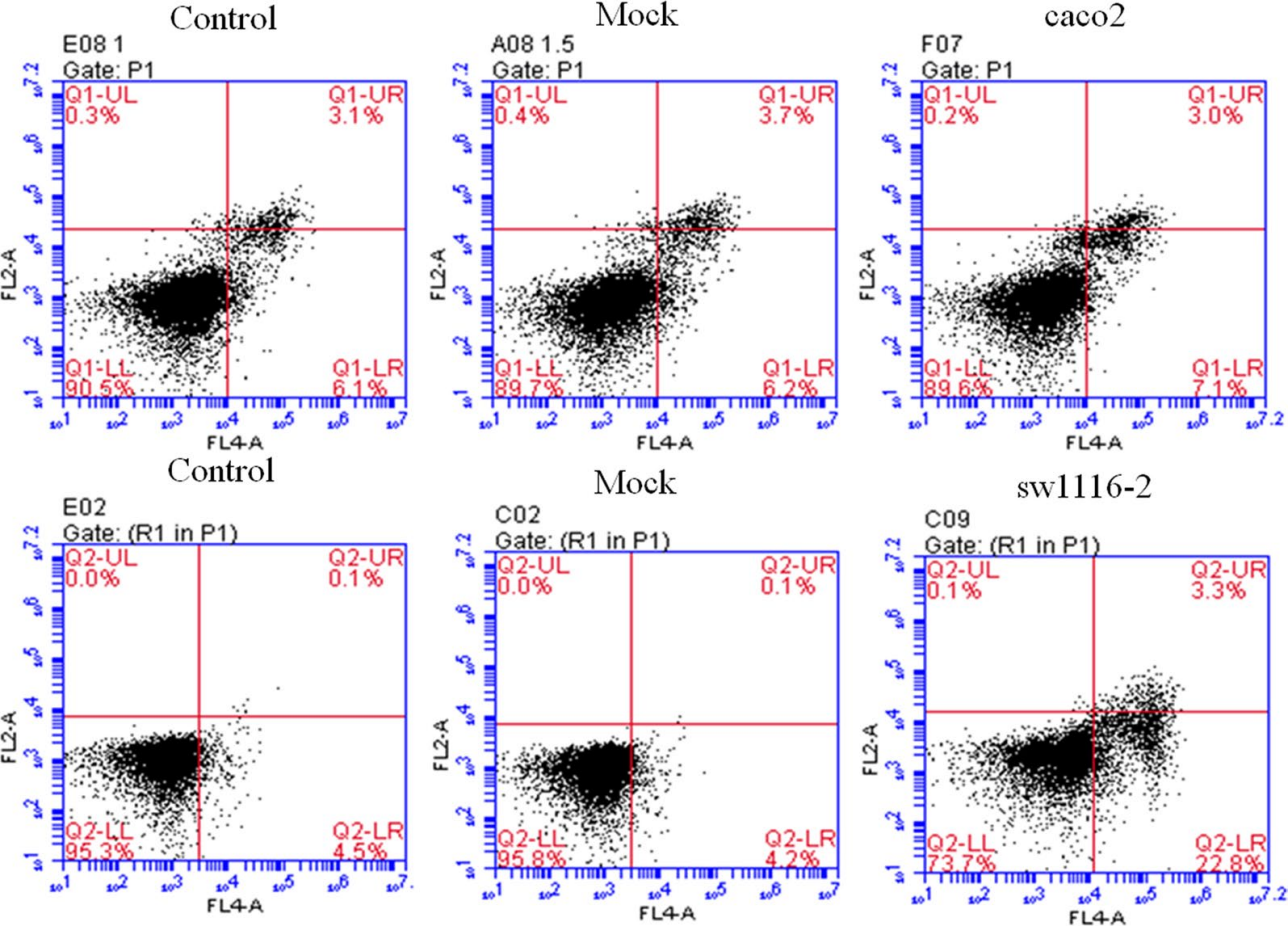
The effect of SFRP1 knockdown on apoptosis of caco-2 cells and the effect of SFRP1 overexpression on apoptosis of sw1116 cells. Apoptosis was assessed using the Annexin V-FITC Apoptosis Detection Kit

**Fig. 4:**
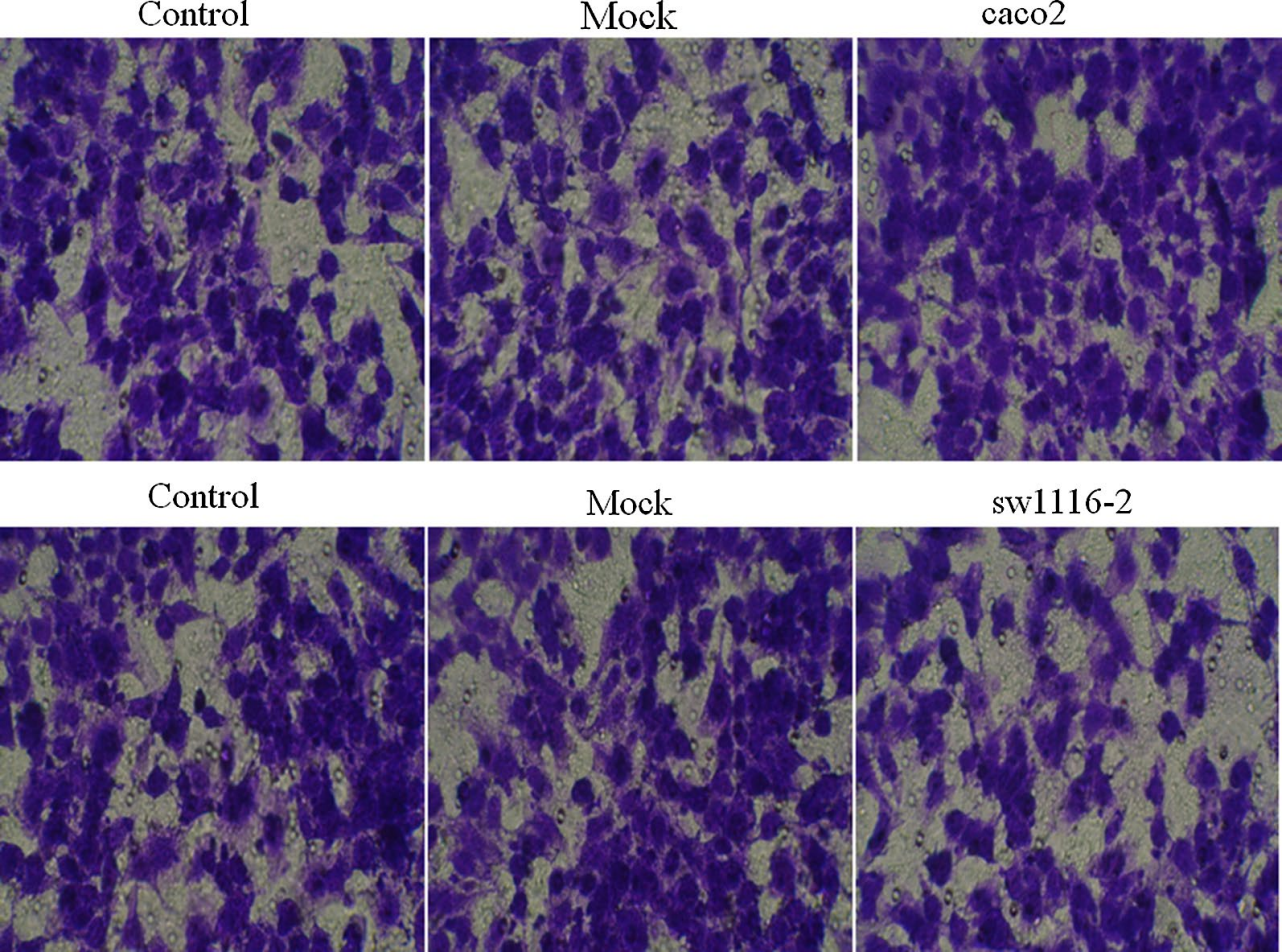
Cell migration analysis of SFRP1 knockdown in caco-2 cells and SFRP1 overexpression in sw1116 cells. Caco-2 cells treated with SFRP1 knockdown and sw1116 cells overexpressing SFRP1 were seeded in the upper chamber of transwell filters. After 24 h incubation, the top of the filters were scraped and cells that Matrigel-invaded through the filters were fixed and stained. Images were representative of invaded cells of one field

**Fig. 5:**
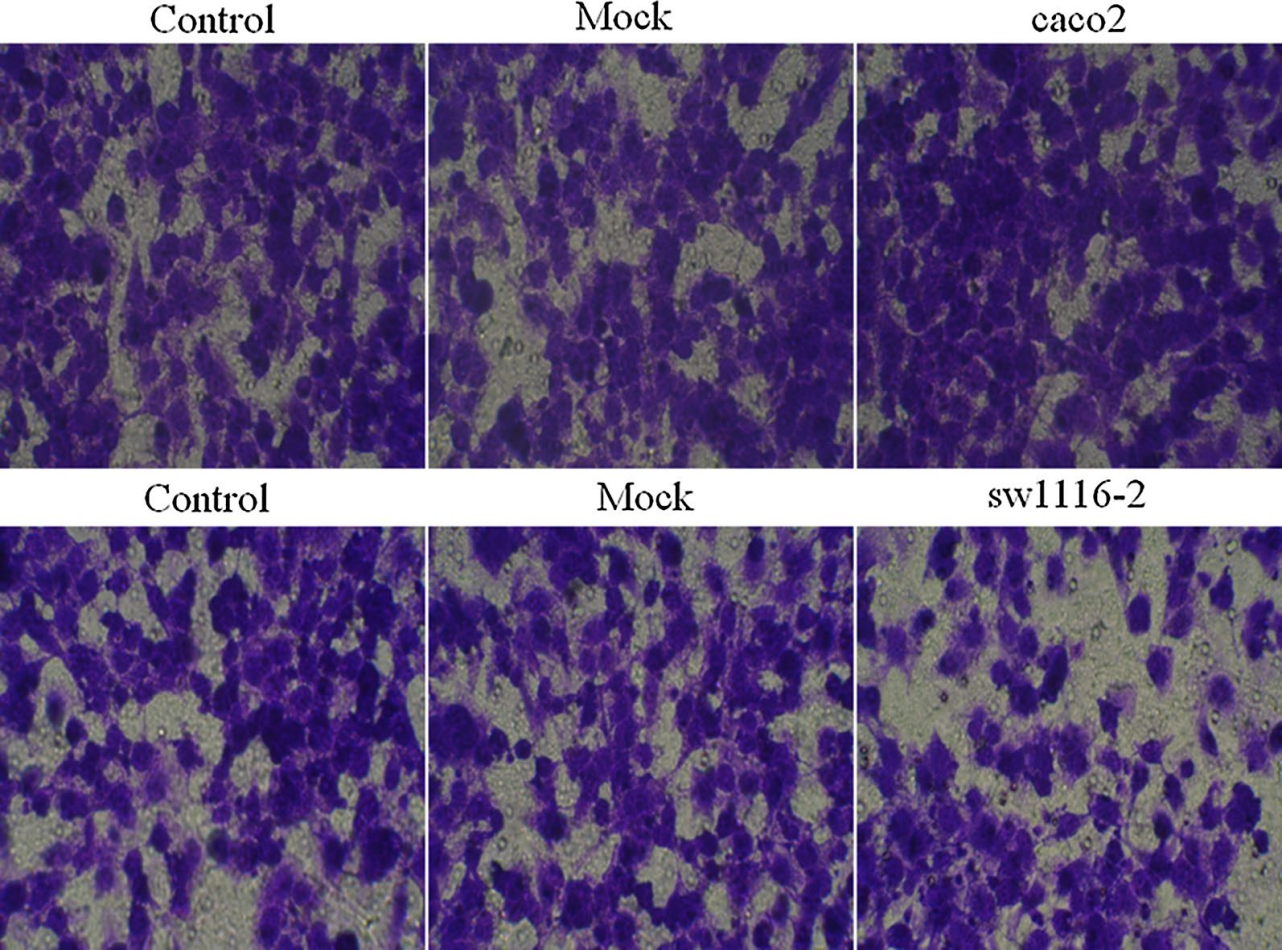
Cell invasion analysis of SFRP1 knockdown in caco-2 cells and SFRP1 overexpression in sw1116 cells. SFRP1 knockdown caco-2 cells and sw1116 cells overexpressing SFRP1 were seeded in the upper chamber of transwell filters. After 24 h incubation, the top of the filters was scraped and cells that Matrigel-invaded the filters were fixed and stained. Representative images of invading cells in one field were shown. Invading cell number was counted in five random fields under a microscope

We performed cell apoptosis analysis to test whether SFRP1 overexpression in sw1116 cell and knockdown of SFRP1 in caco-2 cell affect cell apoptosis. We introduced Control (Normal caco-2 cells or sw1116 cells), Mock (Mock-vehicle treatment of caco-2 cells or sw1116 cells), caco-2 (knockdown of SFRP1) and sw1116 (overexpression of SFRP1). SFRP1 overexpression in sw1116 cells resulted in a significant increase in apoptosis compared with Control and Mock groups (Fig. 3). However, knockdown of SFRP1 in caco-2 cells did not significantly affect apoptosis (Fig. 3). In order to evaluate whether overexpression of SFRP1 in sw1116 cells and SFRP1 knockdown in caco-2 cells affect cell behavior, we used transwell chambers to assess the migration potential of these cells in vitro. sw1116 cells overexpressing SFRP1 showed a significant reduction in migration compared with Control group and Mock group in transwell migration assays (Fig. 4), suggesting that SFRP1 overexpression could effectively suppress CRC cell migration. On the other hand, knockdown of SFRP1 in caco-2 cells lead to enhanced migration in comparison to Control group and Mock group (Fig. 4). Cell invasion analysis demonstrated that overexpression of SFRP1 in sw1116 cells significantly suppressed invasion compared with Control group and Mock group, whereas knockdown of SFRP1 in caco-2 cells resulted in significant enhancement of invasion (Fig. 5).

### Effects of overexpression and knockdown of SFRP1 on CRC cell proliferation

**Fig. 6:**
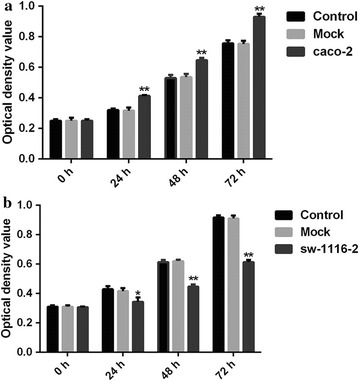
Cell proliferation analysis of SFRP1 knockdown in caco-2 cells and SFRP1 overexpression in sw1116 cells. **a** CCK8 method examined the proliferation of SFRP1 knockdown caco-2 cells at 0, 24, 48, and 72 h. **b** CCK8 method examined the proliferation of sw1116 cells overexpressing SFRP1 at 0, 24, 48, and 72 h

To test whether overexpression of SFRP1 in sw1116 cells and knockdown of SFRP1 in caco-2 cells affect cell proliferation, we introduced Control (Normal caco-2 cells or sw1116 cells), Mock (Mock-vehicle treatment of caco-2 cells or sw1116 cells), caco-2 (knockdown of SFRP1) and sw1116 (overexpression of SFRP1) and evaluated cell proliferation at different time points (0, 24, 48 and 72 h) (Fig. 6). There were no significant differences in cell proliferation among three treatment groups at 0 h. However, knockdown of SFRP1 in caco-2 cells significantly increased cell proliferation compared to Control group and Mock group between 24 and 72 h. There was no significant difference in cell proliferation between Mock group and Control group. On the other hand, overexpression of SFRP1 in sw1116 cells significantly decreased cell proliferation compared with the Control group and Mock group between 48 and 72 h. However, there was no significant difference between Mock group and Control group. Together, these results indicated that both knockdown of SFRP1 in caco-2 cells could increase the cell proliferation and overexpression of SFRP1 in sw1116 cells suppressed cell proliferation.

### Western blot of Wnt/β-catenin signaling pathway

**Fig. 7:**
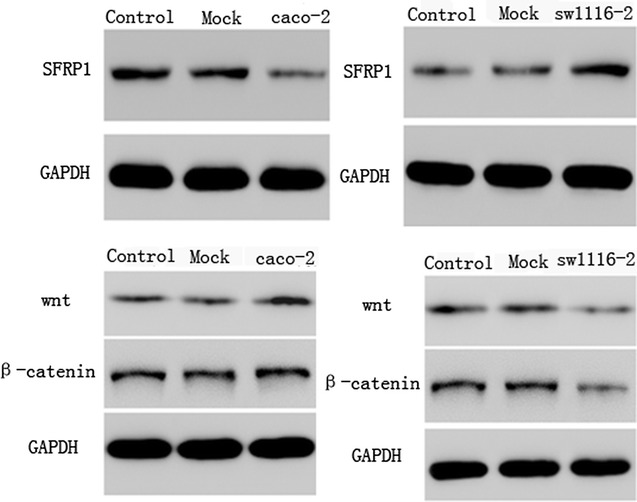
Western blot analysis of Wnt/β-catenin signaling pathway in SFRP1 knockdown caco-2 cells and sw1116 cells overexpressing SFRP1. Control: normal caco-2 cells and sw1116 cells, Mock: mock-vehicle of caco-2 cells and sw1116 cells, caco-2: SFRP1 knockdown in caco-2 cells, sw1116: sw1116 cells overexpressing SFRP1

Western blot was performed to evaluate SFRP1 protein expression in sw1116 cells overexpressing SFRP1 and SFRP1 knockdown caco-2 cells (Fig. 7). In addition, we evaluated protein expression levels of Wnt and β-catenin. In SFRP1 knockdown caco-2 cells at 12 h, there was no significant difference in protein expression levels of Wnt and β-catenin. However, in sw1116 cells overexpressing SFRP1, Wnt and β-catenin protein levels were significantly down-regulated compared with the other two groups.

### Western blot of apoptosis-related biomarkers

**Fig. 8:**
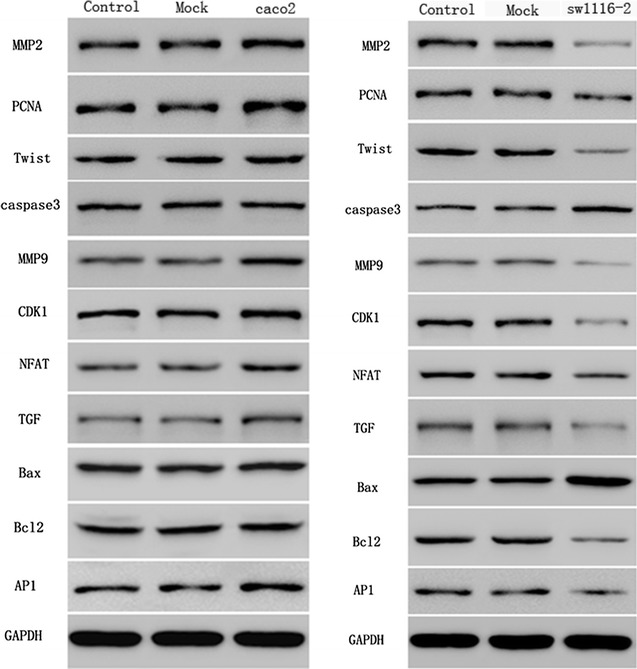
Western blotting was conducted to detect the expression levels of GAPDH, SFRP1, Wnt, β-catenin, MMP2, PCNA, Twist, caspase3, MMP9, CDK1, NFAT, TGF, Bax, Bcl2 and AP1 in SFRP1 knockdown caco-2 cells and sw1116 cells overexpressing SFRP1. Control: normal caco-2 cell and sw1116 cell, Mock: mock-vehicle of caco-2 cell and sw1116 cell, caco-2: SFRP1 knockdown in caco-2 cell line, sw1116: sw1116 cells overexpressing SFRP1

The expression levels of apoptosis-related proteins, including MMP2, PCNA, Twist, caspase3, MMP9, CDK1, NFAT, TGF, Bax, Bcl2 and AP1 were examined by Western blot. GAPDH was used as a loading control. The results showed that SFRP1 knockdown in caco-2 cells had no effects on the expression of these proteins compared with Control group and Mock group (Fig. 8). In sw1116 cells overexpressing SFRP1, the expression levels of PCNA, caspase3, NFAT and AP1 were similar with those of Control group and Mock group (Fig. 8). However, the expression levels of MMP2, Twist, MMP9, CDK1, TGF, and Bcl2 were significantly decreased in sw1116 cells overexpressing SFRP1 compared with Control and Mock group, whereas Bax expression was significantly up-regulated in sw1116 cells overexpressing SFRP1 in comparison to Control and Mock group. It was noteworthy that the protein expression levels of these apoptosis-related factors were similar between Control and Mock groups.

## Discussion

SFRP1 is a glycoprotein known for its ability to negatively modulate the Wnt signalling cascade [11]. Since the Wnt signaling is an important regulator of cancer development and progression, the relation of SFRP1 with tumorigenesis has received much attention from the scientific community. Several recent studies have found dysregulation of SFRP1 expression in human tumors. Suzuki and cowokers demonstrated that the epigenetic loss of SFRP gene function might contribute to the progression of CRC [10]. The study by Ko et al. reported the absence of SFRP1 expression in cervical cancer samples [12]. In addition, overexpression of SFRP1 could induce apoptosis in cervical cancer cells.

In the present study, we detected the expression of SFRP1 in CRC tissues and six cell lines (sw-480, sw1116, caco-2, ht-29, colo-205, and hct-116), and evaluated the effect of SFRP1 on CRC cell proliferation, invasion, migration, and apoptosis. The results demonstrated that SFRP1 expression was dramatically decreased in the primary CRC tissues compared with the corresponding pericarcinomatous tissues, which was consistent with previous findings [7–9]. SFRP1 expression was different between the six CRC cell lines, with the lowest expression level in sw1116 cells and the highest expression level in caco-2 cells. Overexpression of SFRP1 in sw1116 cells resulted in a significantly increase in apoptosis compared with Control and Mock groups. However, knockdown of SFRP1 in caco-2 cells had no effects on apoptosis compared with Control and Mock groups. Overexpression of SFRP1 in sw1116 cells lead to decreased migration compared with Control and Mock groups, while knockdown of SFRP1 in caco-2 cells significantly enhanced migration. Invasion analysis obtained similar findings. Moreover, overexpression of SFRP1 in sw1116 cells significantly suppressed cell proliferation compared with Mock and Control groups up to 72 h. and knockdown of SFRP1 in caco-2 cells resulted in opposite results. We next examined the protein expression level of the Wnt signaling pathway, finding that overexpression of SFRP1 in sw1116 cells significant down-regulated protein expression of the key factors within the Wnt signaling pathway but knockdown of SFRP1 in caco-2 cells did not influence the expression of these proteins. In addition, Western blotting analysis showed that overexpression of SFRP1 in sw1116 cells group remarkedly decreased the protein expression levels of apoptosis-related factors, including MMP2, MMP9, Twist, CDK1, TGF, and Bcl2, but up-regulated the protein expression level of Bax.

Previous studies demonstrated that restoration of SFRP1 and SFRP2 expression in cervical cancer cell lines led to the suppression of cancer cell proliferation, transformation and invasion. These studies also confirmed the cancer suppressor properties of SFRP1 in vivo using a xenograft animal model. The study by Cheng et al., obtained similar results, showing that SFRP2 suppressed the growth of tumors induced by inoculation of gastric cancer cell line MKN-45 in nude mice [13]. In addition, Uren and coworkers demonstrated that activation of the canonical Wnt pathway was necessary and sufficient to induce the transformation of HPV-immortalized human keratinocytes [14]. Interestingly, 80% of cervical carcinomas with increased β-catenin cytoplasmic and nuclear staining had no mutations in the *β*-*catenin* gene [15]. Activation of the canonical Wnt pathway without frequent β-catenin mutations is an interesting finding. One possible mechanism for this activation could be the up-regulation of Wnts, or the modulation of gene expression by DNA methylation of promoter regions.

## Conclusion

We found that the expression of SFRP1 was down-regulated in primary human CRC and a subset of CRC cell lines. In addition, SFRP1 suppressed proliferation, migration and invasion, and promoted apoptosis of CRC cells in vitro. Our findings may offer a potential therapeutic strategy for CRC via targeting SFRP1. Future in vivo studies are required to further clarify the role of SFRP1 in CRC development and progression.
